# The impact of the aortic cusps fusion pattern and valve disease severity on the aortic wall mechanics in patients with bicuspid aortic valve

**DOI:** 10.1007/s10554-020-01838-0

**Published:** 2020-04-17

**Authors:** Mariusz E. Kalinowski, Mariola Szulik, Szymon Pawlak, Barbara Rybus-Kalinowska, Marian Zembala, Zbigniew Kalarus, Tomasz Kukulski

**Affiliations:** 1 Department of Cardiac, Vascular and Endovascular Surgery and Transplantation, Silesian Center for Heart Diseases, Faculty of Medical Science in Zabrze, Medical University of Silesia, Katowice, Poland; 2 Department of Cardiology, Congenital Heart Diseases and Electrotherapy, Silesian Center for Heart Diseases, Faculty of Medical Science in Zabrze, Medical University of Silesia, Katowice, Poland; 3 Department of Cardiac Surgery, Heart Transplantation and Mechanical Support in Children, Silesian Center for Heart Diseases, Zabrze, Faculty of Medical Science in Zabrze, Medical University of Silesia, Katowice, Poland; 4 Department of Basic Medical Sciences, School of Public Health in Bytom, Medical University of Silesia, Katowice, Poland

**Keywords:** Bicuspid aortic valve, Cusp fusion, Aortopathy, Hemodynamic, Mechanics, Tissue doppler imaging

## Abstract

The ascending aorta dilatation in the bicuspid aortic valve (BAV) patients is often attributed to congenital abnormalities of the aortic wall, but it may be related to hemodynamic disturbances in the course of BAV disease. At present, ascending aortic diameter is used as almost sole but weak predictor of aortic dissection and rupture in BAV. We examined the association between aortic wall mechanics and severity of aortic valve disease including different cusps fusion patterns using conventional echocardiography and tissue Doppler imaging (TDI). We prospectively studied 106 BAV patients: 72 with right-left (R-L) coronary cusp fusion were matched 1:1 to 34 patients with right-noncoronary (R-N) cusp fusion obtaining 34 pairs of patients. Peak systolic radial velocity and acceleration of the ascending aortic wall, measured by TDI, were used as an index of hemodynamic stress imposed on the aorta. Paired analysis showed higher aortic wall radial velocity (4.71 ± 1.61 cm/s vs. 3.33 ± 1.44 cm/s, p = 0.001) and acceleration (1.08 ± 0.46 m/s^2^ vs. 0.80 ± 0.34 m/s^2^, p = 0.015) in-R-L compared to R-N fusion. Pearson correlation showed association of ascending tubular aortic diameter with age (r = 0.258, p = 0.012), weight (r = 0.323, p = 0.001), peak aortic valve gradient (r = 0.386, p = 0.0001), aortic root diameter (r = 0.439, p < 0.0001), and R-N fusion pattern (r = 0.209, p = 0.043). Aortic root diameter was related to male gender (r = 0.296, p = 0.003), weight (r = 0.381, p = 0.0001), ascending aortic diameter (r = 0.439, p < 0.0001), and severity of aortic regurgitation (r = 0.337, p = 0.0009). Regional differences in aortic wall motion between different BAV cusp fusion patterns and association of aortic diameters with the severity of aortic valve disease, both suggest a deleterious hemodynamic impact of cusp fusion patterns and aortic valve dysfunction on ascending aortic wall. Assessment of aortic hemodynamic by TDI is feasible and could be potentially used to improve prediction of acute aortic complications, thus helping to establish optimal timing of aortic surgery in BAV patients.

## Introduction

Bicuspid aortic valve (BAV) is the commonest congenital heart defect in adults, occurring in 1–2% of the population, more frequently in men [1]. BAV is associated with an increased incidence of aortic stenosis and regurgitation, coarctation of the aorta, and ascending aortic dilatation, which may lead to aortic dissection and rupture [2, 3]. Familial clustering of BAV is observed, consistent with autosomal dominant mode of inheritance with incomplete penetration [4].

Ascending aortic dilatation and aneurysm formation is a characteristic feature of BAV. The pathogenesis of aortopathy in BAV is not fully understood [5]. Early echocardiographic studies have reported that ascending aortic dilatation is out of proportion to the severity of aortic valve disease, suggesting intrinsic abnormalities of the aortic wall [6, 7]. The results of a subsequent, larger echocardiographic and magnetic resonance imaging (MRI) flow sensitive studies on BAV, suggest that disturbed flow generated by abnormal aortic valve imposes an increased hemodynamic load on the ascending aortic wall leading to the progressive aortic dilatation [8, 9].

A preliminary study demonstrated feasibility of tissue Doppler imaging (TDI) in the assessment of aortic hemodynamic. Increased hemodynamic stress imposed on the ascending aortic wall has been shown in bicuspid aortic valve disease but not in tricuspid stenotic aortic valve [10]. We hypothesized that BAV fusion pattern is associated with hemodynamic stress imposed on the ascending aorta, which can be assessed by TDI. The relationship between severity of aortic valve disease and dilatation of the ascending aorta was also studied.

One of the main problems of BAV is poor prediction of ascending aorta complications like aortic dissection and rupture. So far, mainly ascending aortic diameter was used to anticipate such complications, but with a poor predictive value, thus more parameters are needed for better aortic surveillance in BAV.

## Materials and methods

### Subjects

The study group consisted of 106 BAV patients seen at our institution (mean age, 46 ± 16 years; 15 women). Echocardiographic examinations, including TDI acquisition were performed prospectively in all patients between December 2006 and February 2015.

Exclusion criteria were; moderate to severe valvular and congenital heart disease other than BAV, aortic dissection or coarctation, previous myocardial infarction or cardiac surgery, Marfan’s syndrome, atrial fibrillation, and permanent RV pacing. BAV cusp fusion patterns were determined by two-dimensional echocardiography.

Seventy two patients with right and left coronary cusp fusion were matched 1:1 to 34 patients with right and noncoronary cusps fusion for age, gender, the severity of aortic stenosis and regurgitation, the diameter of aortic root and mid-ascending aorta, and systolic blood pressure (Table 1). As a result, 34 matched pairs of patients were obtained. After matching, groups of patients with different patterns of BAV cusp fusion were comparable (p = NS) with respect to the above-mentioned and remaining variables (Table 2).

**Table 1:** Clinical and echocardiographic parameters in bicuspid aortic valve patients with right-left coronary and right-noncoronary cusp fusion

Variables (n = 106)	All patients (n = 106)	Right-left coronary cusp fusion (n = 72)	Right-noncoronary cusp fusion (n = 34)	P value
Age (years)	45.4 ± 15.9	44.2 ± 15.4	48.2 ± 16.7	0.253
Gender: male, n (%)	91 (85%)	61 (84%)	30 (88%)	0.852
Height (cm)	173.1 ± 8.43	173.2 ± 8.92	173.1 ± 7.30	0.924
Weight (kg)	80.5 ± 10.8	80.1 ± 10.8	81.8 ± 10.9	0.507
Systolic blond pressure (mmHg)	129.4 ± 20.6	131.7 ± 20.4	126.3 ± 20.8	0.242
Diastolic blond pressure (mmHg)	76.7 ± 14.1	77.2 ± 14.9	75.7 ± 12.5	0.681
Aortic Root diameter (mm)	40.1 ± 7.21	40.3 ± 6.97	39.5 ± 7.61	0.574
Ascending aortic diameter (mm)	41.7 ± 6.44	40.8 ± 6.04	43.5 ± 6.77	0.046
AV peak gradient (mmHg)	17.3 ± 13.1	15.7 ± 11.5	21.0 ± 15.03	0.035
AV mean gradient (mmHg)	9.55 ± 7.85	8.52 ± 6.83	11.3 ± 9.28	0.048
Aortic regurgitation: ≥ moderate	61 (57%)	40 (55%)	21 (61%)	0.694
LVEF (%)	54.2 ± 5.34	54.3 ± 4.9	54.0 ± 5.8	0.983
Velocity of aortic wall by TDI (cm/s)	4.04 ± 1.60	4.43 ± 1.66	3.33 ± 1.44	**0.005**
Acceleration of aortic wall by TDI (m/s^2^)	0.96 ± 0.45	1.04 ± 0.48	0.80 ± 0.34	**0.010**

Bold numbers indicate significance at P < 0.05

*AV* aortic valve, *LVEF* left ventricular ejection fraction, *TDI* tissue Doppler imaging, ≥ moderate: moderate to severe

**Table 2:** Clinical and echocardiographic parameters in  matched groups of patients with bicuspid aortic valve

Variables (n = 106)	Right-left coronary cusp fusion (n = 34)	Right-noncoronary cusp fusion (n = 34)	P value
Age (years)	45.2 ± 15.0	48.2 ± 16.7	0.523
Gender: male, n (%)	30 (88%)	30 (88%)	1.000
Height (cm)	173.2 ± 8.28	173.1 ± 7.30	0.707
Weight (kg)	80.1 ± 10.3	81.8 ± 10.9	0.610
Systolic blond pressure (mmHg)	128.9 ± 16.0	126.3 ± 20.8	0.606
Diastolic blond pressure (mmHg)	76.9 ± 13.5	75.7 ± 12.5	0.955
Aortic Root diameter (mm)	39.5 ± 7.20	39.5 ± 7.61	0.820
Ascending aortic diameter (mm)	42.3 ± 6.23	43.5 ± 6.77	0.410
AV peak gradient (mmHg)	19.5 ± 14.2	21.0 ± 15.0	0.749
AV mean gradient (mmHg)	10.6 ± 8.74	11.3 ± 9.28	0.712
Aortic regurgitation: ≥ moderate	20 (58%)	21 (61%)	1.000
LVEF (%)	54.1 ± 5.0	54.0 ± 5.8	0.995
Velocity of aortic wall by TDI (cm/s)	4.71 ± 1.61	3.33 ± 1.44	**0.001**
Acceleration of aortic wall by TDI (m/s^2^)	1.08 ± 0.46	0.80 ± 0.34	**0.015**

Bold numbers indicate significance at P < 0.05

The patients with different cusp fusion pattern were matched for age, gender, aortic root and ascending aortic diameter, peak aortic valve gradient, severity of aortic regurgitation, and systolic blood pressure

*AV* aortic valve, *LVEF* left ventricular ejection fraction, *TDI* tissue doppler imaging, ≥ moderate: moderate to severe

### Echocardiography

Echocardiographic examinations were performed with a commercially available system (Vivid 5, Horten, Norway and Vivid E9, GE Healthcare) equipped with a 2.5- and 3.5 MHz transducers, respectively, by the single experienced echocardiographer (M.E.K), in patients lying on supine using both left and right lateral position.

Left ventricular EF was calculated using the biplane modified Simpson’s rule. The inner diameter of the aortic root and ascending aorta was measured at end-diastole on two-dimensional echocardiography. Ascending aortic diameter was measured distal to the sinotubular junction, at the point of maximal aortic dilatation. Images were obtained in the parasternal long-axis view, perpendicular to the long axis of the aorta.

The aortic valve was observed in multiple views, but the diagnosis of BAV and determination of the cusp fusion pattern was based on the parasternal short-axis view showing only two aortic cusps in systole and diastole. The severity of aortic stenosis and aortic regurgitation was determined by standard methods as recommended by the EACVI/ASE [11].

### Quantification of the aortic wall function

A color doppler tissue imaging data of the mid-ascending anterior aortic wall were collected in the parasternal long-axis view, perpendicular to the long axis of the aorta, at a high frame rate (mean: 177 frames/s) and the aliasing velocity of 16 cm/s. A color tissue doppler images were stored digitally and subsequently transferred to a workstation (EchoPac 6.4.2, GEVingmed Ultrasound 2003) for subsequent off-line analysis.

**Fig. 1:**
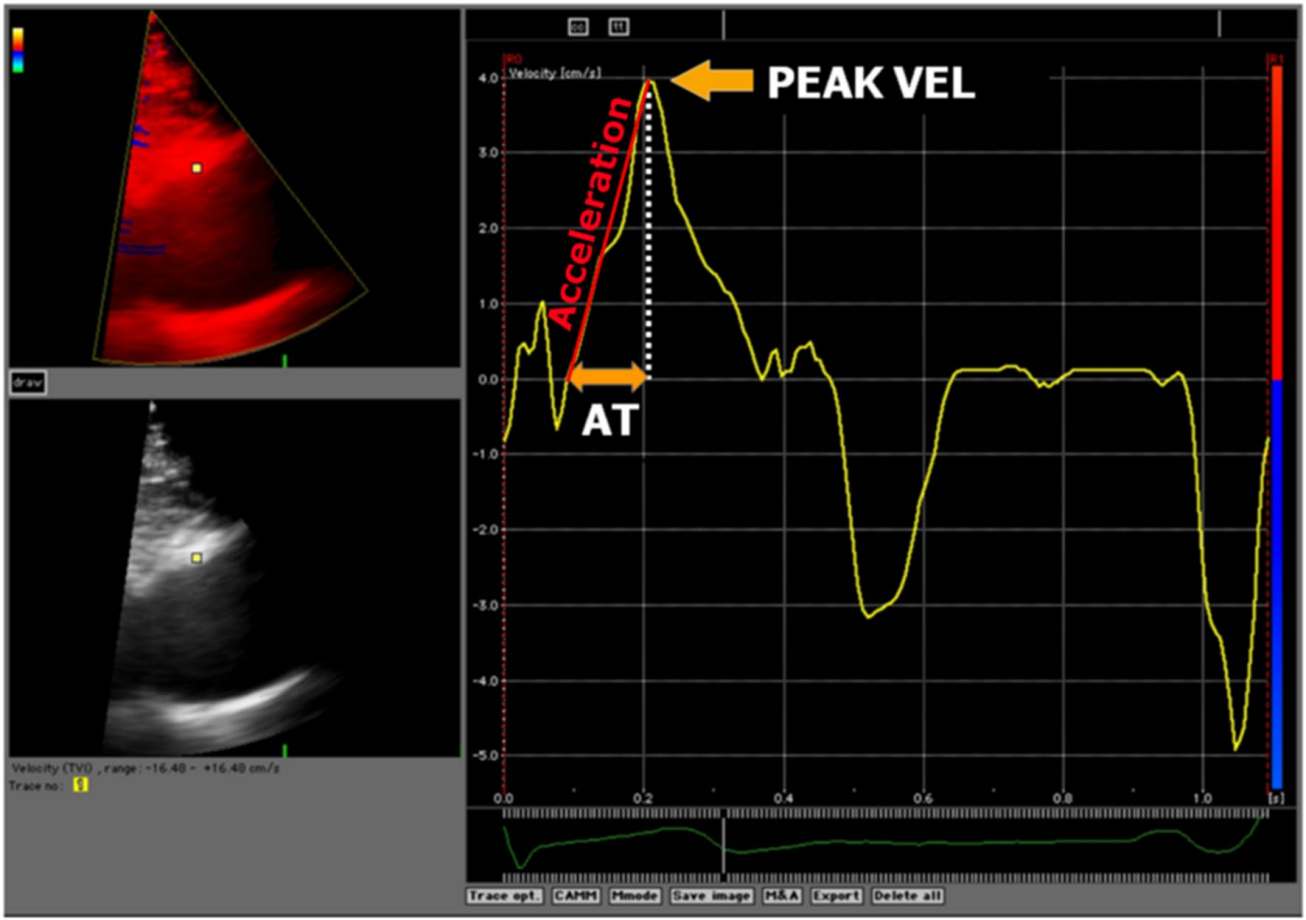
Representative radial velocity curve of the anterior ascending aortic wall recorded by tissue Doppler imaging. Velocity curve was obtained from two-dimensional, color tissue Doppler image of the ascending aorta in the parasternal long-axis view. The single arrow indicates peak systolic velocity of the anterior aortic wall. Oblique red Line represents acceleration. *AT* acceleration time

A 3 × 3 pixel region of interest was placed over the anterior wall of the mid-ascending aorta, 3 cm above the aortic valve. Manual tracking of the sample volume was performed on a frame-by-frame basis throughout the cardiac cycle. Velocity curves were constructed and peak systolic velocity and acceleration were measured and averaged from three consecutive cardiac cycles (Fig. 1).

### Statistical analysis

Continuous variables are presented as a mean ± SD and categorical variables as the numbers and percentages. An optimal matching for propensity score was used to match BAV patients with right-left coronary cusp fusion to patients with right-non coronary fusion. Comparisons between the matched groups of patients were made with Mann–Whitney *U* test. Pearson correlation coefficients were used to evaluate correlations between aortic diameters and other parameters.

The distributions of transvalvular aortic gradients were skewed and therefore log-transformed for correlation analysis. Statistical calculations were performed with SAS software version 9.4 for Windows (SAS Institute Inc., Cary, NC, USA) and SAS macros for optimal matching created by the authors from Mayo Clinic (USA) [12]. Two-tailed P values < 0.05 were considered statistically significant.

An informed consent was obtained from all the subjects. All study protocols were approved by the Institutional Review Board.

## Results

An increased severity of the aortic stenosis, more dilated ascending aorta, but a lower peak systolic radial velocity and velocity acceleration of the anterior ascending aortic wall were observed in patients with right-noncoronary versus right-left coronary cusp fusion (Table 1). Patients with different cusps fusion were matched for clinical and echocardiographic parameters showing that velocity and acceleration of the aortic wall were still higher in patients with right-left coronary cusp fusion (Table 2).

Correlation analysis showed that ascending tubular aortic diameter was significantly and positively associated with age (r = 0.258, p = 0.012), weight (r = 0.323, p = 0.001), log peak aortic valve gradient (r = 0.386, p = 0.0001), aortic root diameter (r = 0.439, p < 0.0001), and right-noncoronary fusion pattern (r = 0.209, p = 0.043). Aortic root diameter was significantly and positively correlated with male gender (r = 0.296, p = 0.003), weight (r = 0.381, p = 0.0001), height (r = 0.299, p = 0.003), ascending aortic diameter (r = 0.439, p < 0.0001), and severity of aortic regurgitation (r = 0.337, p = 0.0009).

## Discussion

The results of our study suggest that local hemodynamic load imposed on the ascending aortic wall depends on the type of BAV fusion and severity of aortic valve disease and that TDI appears to be feasible for assessment of aortic hemodynamic. Aorta is the largest, most elastic artery. These properties allow for accommodation of extensive volume of pulsating blood flow with low resistance, thus diminishing left ventricular afterload. Aortic wall elasticity diminished progressively with increasing distance from the heart due to an increasing amount of collagen in relation to elastin fibers. This feature, along with elastic aortic recoil in diastole provides damping function (Windkessel effect) leading to protective nearly steady blood flow to end organs [13]. The mechanical function of the aorta is determined by its particular, three-layer structure. The main components responsible for aortic mechanical properties are situated primarily in tunica media, the thickest aortic layer. These are elastin fibers aligned in concentric elastic lamellae—the major determinants of aortic distensibility, collagen fibers providing tensile strength, as well as smooth muscle cells. A three-dimensional, highly organized structure is built by these constituents [14].

Elastic properties of aortic wall could be screened noninvasively by imaging techniques. This could be done by assessing changes in aortic volume in relation to pulse pressure (distensibility) or by measuring the time of blood flow wave traveling along the aorta (pulse wave velocity) [15].While ultrasonography could be used here, advanced MRI is capable of simultaneously investigating these elastic parameters and hemodynamic behavior of the aorta [16].

There is a controversy regarding the pathogenesis of ascending aortic dilatation in BAV patients [5, 9]. One theory suggests that developmental abnormalities of both aortic valve and the ascending aortic wall are present in BAV. The common embryological origin of these structures from the neural crest [17], the occurrence of aortic dilatation in first-degree relatives of BAV patients [18], presence of aortic dilatation out of proportion to the severity of aortic valve disease in early echocardiographic studies, [6, 7], as well as ongoing ascending aortic dilatation after aortic valve replacement [19] make this explanation probable [20]. Another theory is that disturbed aortic blood flow due to congenitally malformed BAV increases local hemodynamic forces which contribute to impairment of aortic media via mechanotransduction [8, 9].

The seminal study by Robicsek et al. on excised human aortic roots with BAV in the left heart simulator revealed structural malformations and reduced anatomic area despite apparently normal function of BAVs. These valve abnormalities were associated with formation of distinct large flow “vortices” that were not limited to the aortic sinuses, as in the case of tricuspid aortic valve, but they moved distally, increasing hemodynamic stress imposed primarily on the greater curvature of the ascending aorta [21].

Further advances in research of aortic hemodynamic were achieved by introducing three- and four- (time resolved)-dimensional flow MRI [22]. In the recent flow-sensitive MRI studies, the pattern of aortic flow has been shown directly, which confirmed the above-mentioned observations. Extensive right-handed helical flow prevailed in BAV, with markedly asymmetric pattern of both postvalvular and distal flow, leading to increased aortic wall shear stress locally [23, 24].

Previous studies on BAV’s have shown spatial differences concerning abnormalities of the ascending aortic media. Immuno-histochemistry of the aortic wall specimens in BAV patients revealed a decrease in smooth muscle cells and pathological changes in stress-related matrix protein composition which was observed especially in the areas where hemodynamic stress was expected to be higher (convexity of the ascending aorta) [25, 26].

A preliminary study using echocardiography for the assessment of ascending aortic hemodynamic was performed in patients with aortic stenosis, by determining the peak radial velocity of the aortic wall using TDI. An increased hemodynamic load imposed on the aortic wall was reported in bicuspid vs tricuspid valve patients. Furthermore, an asymmetric distribution of hemodynamic forces was recognized only in BAV patients, showing increased stress placed on the anterior aortic wall [10].

To the best of our knowledge, our study is the first to examine aortic hemodynamic in patients with different BAV cusp fusion pattern using TDI technique. One would expect that the higher blood flow induced by BAV would account for the increased systolic outward motion of the aortic wall. In our study, aortic blood flow has not been shown directly; instead, radial aortic wall motion was assessed which was assumed to be influenced by the hemodynamic burden caused by abnormal aortic flow. The influence of different patterns of BAV cusp fusion on aortic hemodynamic can be accurately determined only after adjustment for confounding factors that may affect aortic wall motion. Accordingly, we have matched right-noncoronary with right-left coronary cusp fusion patients not only for age and gender, but also for the aortic root and ascending aortic diameter, systolic blood pressure, and the severity of aortic stenosis and regurgitation.

**Fig. 2:**
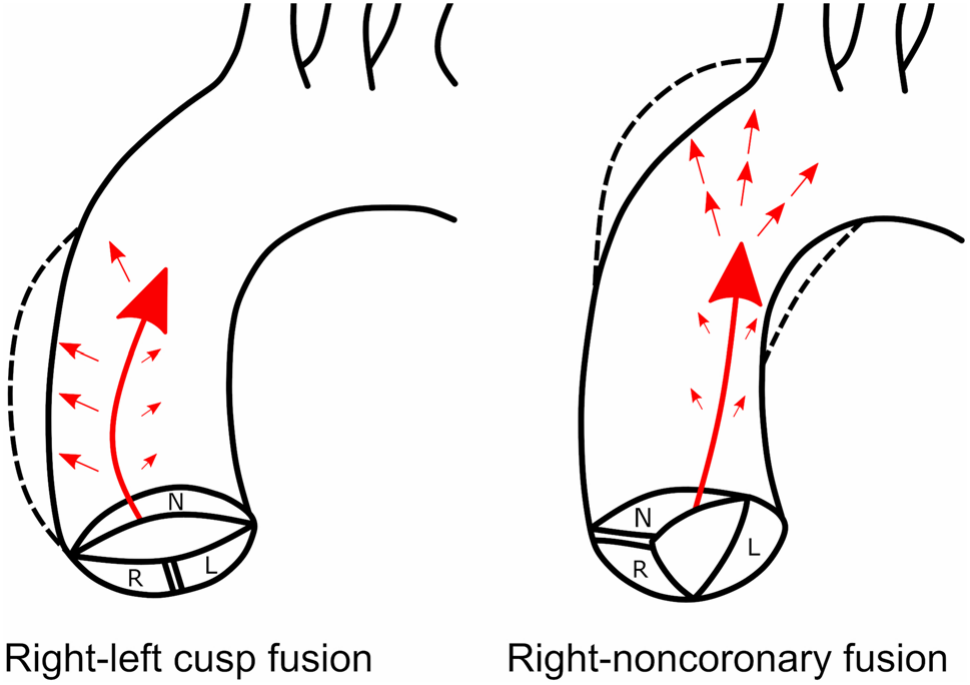
Scheme of the of the bicuspid aortic valves with different type of cusp fusion. In right to left cusp fusion (see left) blood flow in aorta is directed more anteriorly and to the right impinging proximal ascending aortic wall (dilatation of mainly aortic sinuses), while in R–N fusion (see right) blood travels posteriorly and to the left, thus increasing hemodynamic load on the aortic wall more distally (dilatation of mid-ascending aorta and even aortic arch). Such hemodynamic characteristics are directly observed by MRI and are also reflected by the results of our study

The results of our study on BAV suggest that blood flow in the right-left coronary cusp fusion is directed through the aortic valve mainly towards the anterior wall of the ascending aorta, which is more susceptible to hemodynamic stress. This may result in the progressive damage to the aortic wall, and finally aortic dilatation (Fig. 2). Previous study demonstrated a greater aortic root diameter and the degree of morphological wall abnormalities in BAV patients with right-left coronary cusp fusion pattern [27].

In the recent BAV study histopathological changes in the aortic media were assessed together with the patterns of ascending aortic wall shear stress distribution showed by flow sensitive MRI. Degeneration of elastin and disturbances of matrix regulatory protein were found mainly in aortic areas of increased wall shear stress, suggesting a cause-and-effect relationship [28].

Recent MRI studies assessing aortic flow in BAV patients confirmed that in patients with right-left coronary cusp fusion a higher blood flow was directed towards the antero-lateral wall of the ascending aorta contrary to right-noncoronary fusion where greater stress was found at posterior aortic wall [23, 29]. Similarly, our TDI study revealed increased systolic radial velocity and acceleration of the anterior aortic wall in BAV patients with right-left coronary cusp fusion contrary to right-noncoronary fusion. This suggests that screening for ascending aortic hemodynamic with this imaging technique seems feasible.

At present, ascending aortic diameter is used as almost sole but weak predictor of aortic dissection and rupture. BAV morphological phenotypes emerge as a new predictor of aortic remodeling. Aortic stenosis was observed to be associated with dilatation of ascending tubular aorta, while aortic regurgitation was correlated with dilatation of aortic sinuses [30]. Moreover R-N cusp fusion was associated with dilatation of predominantly ascending tubular aorta while R-L fusion was related to dilatation of aortic sinuses—root phenotype that was the most significant predictor of faster ascending aortic growth rate [31].

Research is ongoing to find new biomarkers predicting aortic complications in BAV predominantly among imaging techniques and blood parameters. Regarding imaging techniques there are initial attempts to translate advanced ultrasonography techniques used for heart studies to examine aortic wall biomechanics e.g. aortic wall strain with speckle tracking imaging introduced for screening ascending aortic stiffness [32] and subsequently used to study elastic aorta properties in BAV [33].

Emerging blood biomarkers of aortic wall damage in BAV are metalloproteinases (MMPs)—proteolytic enzymes capable of degrading extracellular matrix components of aortic media and tissue inhibitors of metalloproteinases (TIMPs),as well as transforming growth factor ß (TGF-ß) and specific microRNAs implicated in pathophysiology of aortic wall remodeling [34].

Genetic abnormalities may also belong here, e.g. mutations in NOTCH1, GATA family and ACTA2 genes which lead to BAV formation and could contribute to structural abnormalities of ascending aortic wall [35].

Aortic stenosis and regurgitation may increase the hemodynamic load imposed on the ascending aortic wall resulting in increased wall remodeling and aortic dilatation. In our study the relation between the severity of aortic valve disease and ascending aortic diameter was shown together with regional differences in aortic wall motion between different BAV cusp fusion patterns which seem to confirm hemodynamic impact of cusp fusion patterns and aortic valve dysfunction on ascending aortic wall. This is in agreement with previous echocardiographic studies in larger numbers of BAV patients showing a similar relation between aortic dilatation phenotype and degree of aortic stenosis and regurgitation [36, 37]. Recent MRI study in BAV patients revealed that the presence of moderate to severe aortic stenosis was associated with increased shear stress and aortic diameter as well as a more extensive helical and eccentric flow pattern in the ascending aorta [24].

### Limitations

The relatively small number of women in our study makes it difficult to apply our results in this group.

Precise blood flow patterns in the aorta could not be directly visualized and elastic properties of the aortic wall were not studied in our exploratory echocardiographic study, but we believe that the relative influence of aortic stiffness on tissue Doppler indices of aortic wall motion should be studied by referred PWV method in gold-standard imaging technique i.e. advanced MRI. By using this imaging modality, elastic aortic properties, together with 3-dimensional disturbances of aortic flow could be described simultaneously. Nevertheless, taking into account costly and not widely available 4-D flow MRI software, using TDI parameters of aortic wall motion could improve the present limited prediction of ascending aortic complications in BAV patients.

## Conclusions

Radial velocity and velocity acceleration of the ascending aortic wall, as assessed by TDI, were found to be associated with the cusps fusion pattern of BAV. This suggests that the hemodynamic stress placed on the ascending aortic wall depends on BAV phenotypes. A quantitative analysis of the ascending aortic wall motion using TDI is feasible and may be useful in the evaluation of aortic hemodynamic burden, as a new potential predictor of acute aortic complications. The severity of BAV stenosis and regurgitation was associated with ascending tubular aorta and aortic root dilatation, respectively, implying that these valve lesions induce hemodynamic impact on the aortic wall. At present, both increased hemodynamic burden induced by the diseased aortic valve and congenital abnormalities of the aortic wall are the most probable cause of progressive ascending aortic dilatation in BAV.

## Clinical perspectives

Our findings suggest that TDI could be included in future BAV studies evaluating the importance of hemodynamic factors, both in the pathogenesis of the ascending aortic dilatation and in pharmacological or surgical treatment. This imaging modality may be useful for predicting aortic dilatation rate more precisely, which may help to better define the frequency of follow-up visits and the time of prophylactic surgical intervention on the aorta. Although our results should be confirmed by other studies, they appear to provide supporting evidence that hemodynamic stress plays an important role in the pathogenesis of BAV aortopathy. Tissue Doppler is a promising tool for the assessment of hemodynamic forces imposed on ascending aortic wall.

## Abbreviations

BAV: Bicuspid aortic valve
TDI: Tissue Doppler imaging
